# Epidemiological analysis of *Dirofilaria immitis* (Spirurida: Onchocercidae) infecting pet dogs (*Canis lupus familiaris*, Linnaeus, 1758) in Baixada Fluminense, Rio de Janeiro

**DOI:** 10.3389/fvets.2024.1360593

**Published:** 2024-05-02

**Authors:** Viviane Marques de Andrade Vieira, Priscila Pinho da Silva, Érica Tex Paulino, Priscila do Amaral Fernandes, Norma Labarthe, Gilberto Salles Gazêta, Antonio Henrique Almeida de Moraes Neto

**Affiliations:** ^1^Laboratory of Innovations in Therapies, Teaching and Bioproducts, Oswaldo Cruz Institute, Oswaldo Cruz Foundation (LITEB/IOC/FIOCRUZ), Rio de Janeiro, Brazil; ^2^Tropical Medicine Program, Oswaldo Cruz Institute, Oswaldo Cruz Foundation (IOC/FIOCRUZ), Rio de Janeiro, Brazil; ^3^Laborlife Análises Clínicas, Rio de Janeiro, Brazil; ^4^National School of Public Health, Oswaldo Cruz Foundation (ENSP/FIOCRUZ), Rio de Janeiro, Brazil; ^5^Laboratory of Ticks and Other Apterous Arthropods - National Reference Laboratory for Vectors of Rickettsioses, Oswaldo Cruz Institute, Oswaldo Cruz Foundation (LAC/IOC/FIOCRUZ), Rio de Janeiro, Brazil

**Keywords:** *Dirofilaria immitis*, heartworm, zoonosis, epidemiology, pet dog

## Abstract

*Dirofilaria immitis* infection is routinely detected in dogs during veterinary care in tropical and subtropical regions worldwide. Parasitological tests for the detection of this infection are routinely performed only in areas with a high prevalence. Baixada Fluminense, a region in Rio de Janeiro, was considered heartworm-free until local veterinarians began to receive blood exams results indicating the presence of microfilariae (MF). A laboratory database was hence used to collect data from 2017 to 2020 to understand the extent of spread of the parasite in this area. The results of complete blood count analysis and MF or heartworm antigen detection tests conducted on canine samples sent from veterinary clinics in Baixada Fluminense (Magé, Duque de Caxias, Guapimirim, Nova Iguaçu, and São João de Meriti municipalities) were included. In total, the results of 16,314 hematological tests were considered. The overall prevalence of *D. immitis* was 3.4% (554/16,314), considering that only one test result was obtained per animal on the same day. This study is highly relevant because it indicates the spreading geographic distribution of the worms, heightens awareness among local health professionals and the general population, and encourages compliance with prophylactic measures to prevent further spread of parasite.

## 1 Introduction

Some filarioids, such as *Dirofilaria immitis*, *Dirofilaria repens*, *Acanthocheilonema reconditum*, *Oncocherca lupi,* and *Cercopithifilaria grassii*, belonging to the family Onchocercidae (Spirurida), are transmitted by arthropod vectors and cause canine filariasis (1). Worldwide, more than seventy species of the Culicidae family participate in the transmission of *D. immitis* and the main mosquito-vectors are: *Aedes*, *Ochlerotatus*, *Culex*, *Anopheles* (2). Competent vectors ingest microfilariae (MF) when they take a blood meal. In about 10 to 14 days, depending on the environmental temperature, the larvae develop into third stage (L3) and migrate to the head of the mosquito. When the mosquito takes the next blood meal the L3 migrates to the new definitive host. Once the new host is infected the L3 molts to L4 and in approximately 120 days young adults can be found in the pulmonary arteries and right chambers of the heart. Adult males and females’ mate and produce MFs that can be found in the peripheral blood stream approximately in 7 to 9 months (3).

Canine clinical signs are multifactorial. Most dogs are asymptomatic and when they become sick, coughing, weight loss and exercise intolerance are frequent. Severe disease includes signs of congestive right heart failure (4). An update on the South American seroprevalence showed that no infected dog has been reported in Chile and that in the other countries were the infection has been detected, prevalence rates range from 14.41% in Argentina to 1.6% in Colombia, 8.9% in Mexico, 5.5% in Peru and 15.2% in Venezuela (5). The overall prevalence of canine *D. immitis* infection in Brazil was 13.03% (6). *Dirofilaria immitis* canine infection is common in the coastal regions of Brazil, with a high prevalence of 23.1% (7).

In the State of Rio de Janeiro, during the active search for cases of canine heartworm disease, seroprevalence was recorded in some locations in the metropolitan region where no survey had been carried out. In the west zone, a study showed that 21.6% of canines were infected (8); another research showed that laboratories that received samples from different neighborhoods in the city of Rio de Janeiro reported only 7% of nematode infections in dogs (9) and occurrences were reported during veterinary care on Ilha do Governador showing that 14.5% of dogs were infected by *D. immitis* (10).

The Baixada Fluminense region was considered to be indene, until 2004 when a record of a case with a frequency rate of 0.9% in the municipality of Nova Iguaçu (11). After 2017, *D. immitis* has been detected at a higher frequency with autochthonous cases reported in this area (12, 13). The Baixada Fluminense region has recently been recognized as a new focus area for onchocercid infections (13). Undoubtedly, global climate change and anthropogenic actions favor increased human, canine, and mosquito population densities and, thus, the spread of the infection (14).

Traveling with dogs is increasing owing to the easiness. Some families travel with multi-species pets. Although this practice may be incentivized, the associated health issues must not be ignored. One way to counteract these health issues is through good pet care, including preventive measures that undoubtedly impose chemoprophylaxis on *D. immitis*. Therefore, infections monitoring and spreading awareness, particularly in areas without parasite circulation, must be prioritized locally. This study aimed to analyze the epizootiological factors including the prevalence of infections *of D. immitis* in domestic dogs in Baixada Fluminense, Rio de Janeiro, Brazil.

## 2 Materials and methods

### 2.1 Ethical aspects

The study was approved by the Animal Use Ethics Committee of the Oswaldo Cruz Institute/Oswaldo Cruz Foundation (CEUA-IOC-L009/2020) and the Oswaldo Cruz Institute/Oswaldo Cruz Foundation Human Research Ethics Committee (CEP CAAE: 30759620.1.0000.5248).

### 2.2 Study location

The study was performed as a retrospective analysis of the Laborlife Clinical Analysis Laboratory^1^ database from January 2017 through December 2020, including dogs that lived in Baixada Fluminense (total area of 43,696 km^2^; below 200 meters altitude), metropolitan region of the State of Rio de Janeiro. The Atlantic Forest Biome touches the border areas of Baixada Fluminense compromising a vast area of environmental conservation with ecological stations and parks, a semi-humid tropical climate, and the average annual temperature of 24°C.^2^ The municipalities included in this study were Nova Iguaçu (22° 45′33″S, 43° 27′04″W), Magé (22° 39′10″S, 43° 02′26″W), Guapimirim (22° 32′14″S, 42° 58′55″W), Duque de Caxias (22° 47′ 08″S, 43° 18′42″W) and São João de Meriti (22° 48′14″S, 43° 22′22″W).

### 2.3 Data collection

The data was limited to that of blood samples obtained from dogs over 12 months of age to avoid bias due to the long prepatent period of the infection and that collected by attending veterinarians of private clinics or hospitals located in one of the five municipalities of Baixada Fluminense (Metropolitan Rio de Janeiro). The data included: (i) *D. immitis* antigen detection test results (lateral flow immunochromatographic assay – Alere™ Dirofilariasis Ag Test Kit; BioNote, Inc., Republic of Korea, or enzyme immunoassay – SNAP^®^ 4Dx^®^ Plus; IDEXX Laboratories, Westbrook, MN, United States); (ii) results of modified Knott’s test to detect microfilariae (15); and (iii) unexpected findings obtained during blood smear for CBC or hemoparasite investigation. When an infection was detected in a dog using one technique, results from other methods were excluded to avoid duplication. When antigen detection test result was available, it was considered first. Knott’s test results were considered when the antigen test result was unavailable, and blood smear results were considered only when none of the other were available. In these cases, the presence of microfilariae was recorded.

### 2.4 Statistical analysis

Was evaluated the following characteristics were evaluated: the municipality of residence, age (>12 months), sex (male or female), and tests for the detection of adult worms and microfilariae. Pearson’s chi-squared test was used to determine the association between these characteristics and the test results. As some variables had more than two categories, a post-hoc analysis of the adjusted standardized residuals was performed to identify each variable’s specific pairs of associated categories. The *p*-values were adjusted using the Bonferroni method to account for multiple comparisons. All analyses were performed using SPSS Statistics software version 24 (16) with an α significance level of 5%.

## 3 Results

**Figure 1:**
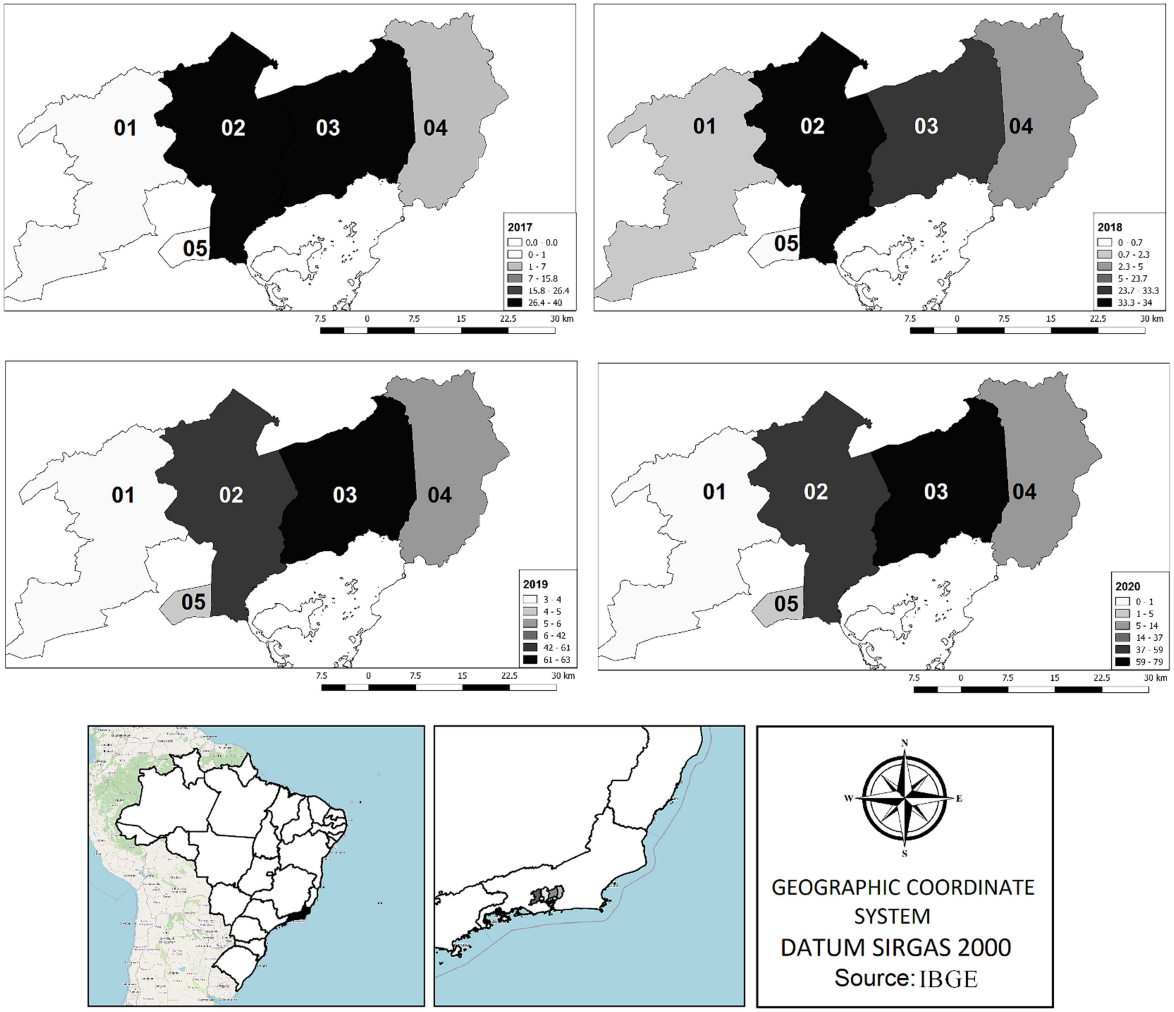
Positive cases of *Dirofilaria immitis* in dogs (aged >12 months) in Baixada Fluminense, Rio de Janeiro State, Southeast Brazil (QGis software version 2.18). 1- Nova Iguaçu, 2- Duque de Caxias, 3- Magé, 4- Guapimirim, 5- São João de Meriti.

**Figure 2:**
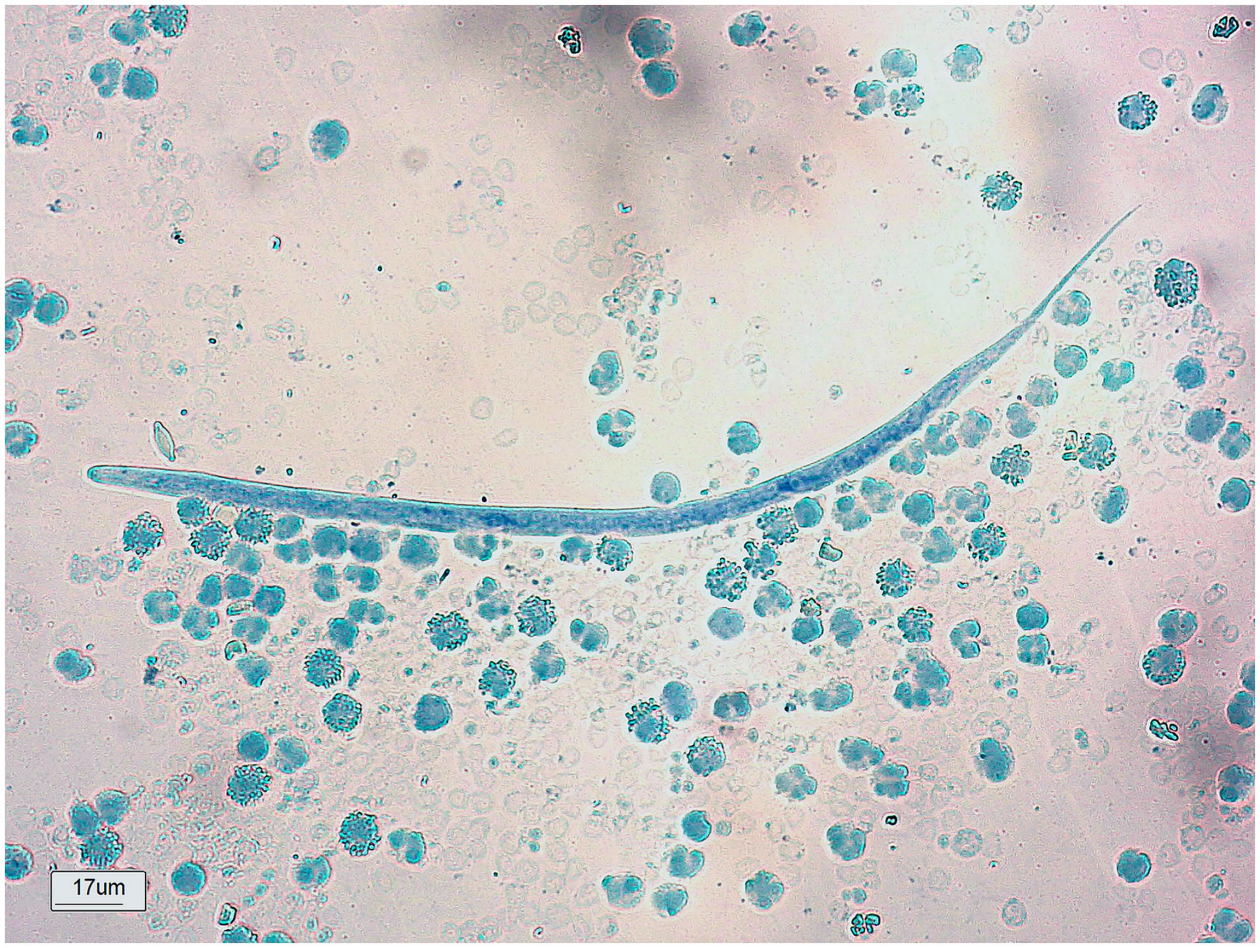
Microfilariae of *Dirofilaria immitis*, detected by the modified Knott’s technique.

The analysis included 16,314 test results, of which 3.4% were positive for *D. immitis* (Table 1). The highest overall prevalence was observed in Magé, where 8.5% (270/3,162) of the dogs tested positive, followed by Duque de Caxias, where 2.5% tested positive (241/9,788) (Table 1). The space–time distribution shown in Figure 1 indicates that these municipalities have remained the same over the years, with a greater number of cases than those in the others. According to antigen tests, 25.3% (62/245) of the dogs were positive for the *D. immitis*, 17.5% (157/897) were positive for the modified Knott’s test, and 2.2% (335/15,172) were positive for unexpected findings of microfilariae (Table 1; Figure 2).

**Table 1:** Epizootiological data associated with the prevalence of *D. immitis* in canines in 2017–2020 in Baixada Fluminense, RJ.

Characteristics	*D. immitis n* (%)		*p*-value	Total *n* (%)
	No	Yes		
**Municipalities**			0.000*	
Magé	2,892 (17.7)^a^	270 (1.7)^b^		3,162 (19.4)
Duque de Caxias	9,547 (58.5)^a^	241 (1.5)^b^		9,788 (60.0)
São João de Meriti	971 (6.0)^a^	5 (0.0)^b^		976 (6.0)
Nova Iguaçu	465 (2.9)^a^	6 (0.0)^b^		471 (2.9)
Guapimirim	1,885 (11.6)^a^	32 (0.2)^b^		1,917 (11.7)
**Age (years)**			0.020*	
1–7	7,591 (46.5)^a^	244 (1.5)^b^		7,835 (48.0)
8–14	3,223 (19.8)^a^	135 (0.8)^b^		3,358 (20.6)
15 or more	337 (2.1)^a^	7 (0.0)^a^		344 (2.1)
Uninformed	4,609 (28.3)	168 (1.0)		4,777 (29.3)
**Sex**			0.000*	
Female	8,165 (50.0)^a^	242 (1.5)^b^		8,407 (51.5)
Male	7,595 (46.6)^a^	312 (1.9)^b^		7,907 (48.5)
**Tests**			0.000*	
Unexpected findings	14,837 (90.9)^a^	335 (2.0)^b^		15,172 (93.0)
*D. immitis* antigen	183 (1.1)^a^	62 (0.4)^b^		245 (1.50)
Modified Knott’s test	740 (4.5)^a^	157 (1.0)^b^		897 (5.50)
**Total**	15,760 (96.6)	554 (3.4)		16,314 (100)

*p-value < 0.05; ^a,b,c^ each letter indicates categories of variables that do not differ at a significance level of 0.05.

The results indicated a significant association between infection and all canine characteristics (Tables 1, 2). Compared to dogs treated at veterinary clinics in Magé, those treated in Duque de Caxias, São João de Meriti, Nova Iguaçu, and Guapimirim were 73% (OR = 0.270; CI95% = 0.226–0.323), 94.5% (OR = 0.055; CI95% = 0.023–0.134), 86.2% (OR = 0.138; CI95% = 0.061–0.312), and 81.8% (OR = 0.182; CI95% = 0.125–0.263) less likely, respectively, to have positive results (Tables 1, 2). Furthermore, dogs treated in São João de Meriti were 79.6% less likely (OR = 0.204; CI95% = 0.084–0.496) to be infected than those treated in Duque de Caxias (Tables 1, 2). Age was also a significant factor, with dogs aged 8–14 years being 30.3% more likely (OR = 1.303; CI95% = 1.052–1.614) to be infected than those aged 1–7 years. Male dogs were 38.6% more likely (OR = 1.386; CI95% = 1.168–1.644) to be infected than female dogs (Tables 1, 2). Moreover, the *D. immitis* antigen test showed 59.6% (OR = 1.596; CI95% = 1.141–2.233) more positive results than the modified Knott’s test (Tables 1, 2).

**Table 2:** The Odds ratio between each variable’s categories differs, referring to the chi-square posthoc test.

Characteristics	OR	CI95%
**Municipalities**		
Magé vs. Duque de Caxias	0.270	0.226–0.323
Magé vs. São João de Meriti	0.055	0.023–0.134
Magé vs. Nova Iguaçu	0.138	0.061–0.312
Magé vs. Guapimirim	0.182	0.125–0.263
Duque de Caxias vs. São João de Meriti	0.204	0.084–0.496
**Age (years)**		
1–7 vs. 8–14	1.303	1.052–1.614
**Sex**		
Female vs. Male	1.386	1.168–1.644
**Tests**		
Unexpected findings vs. *D. immitis* antigen	15.005	11.030–20.411
Unexpected findings vs. Modified Knott’s test	9.396	7.666–11.516
Modified Knott’s test vs. *D. immitis* antigen	1.596	1.141–2.233

OR, odds ratio; CI95%, confidence interval 95%.

## 4 Discussion

According to a previous report using multiplex PCR, at least 93.5% of dogs in the study area were infected with *D. immitis* (13). Hence, in this study MF detected by conventional tests displaying the morphology of the anterior and of the posterior ends in agreement with *D. immitis* description (17, 18) were assumed to be *D. immitis* by the laboratory. This incomplete identification of the larvae morphology can be considered a limitation in this study.

Male dogs were infected more frequently than female dogs. This has been previously reported (19) however, no hypothesis has been proposed to explain this difference (19, 20). Empirical observations have shown that spaying and neutering dogs in the study area are rare, suggesting that females need to be better cared for to avoid unwanted litter and that male dogs are mainly restricted to backyards or sometimes allowed to roam free, as observed elsewhere (19, 20). This human behavioral manner, along with the predisposition of male dogs, suggests that the difference may be attributed to the exposure to infected mosquitoes instead of the sex.

The frequency of infections among older dogs (8–14 years) may have been higher than that among younger dogs by chance. When moderately challenged as observed (frequency of 3.4%), the longer the exposure to the vectors, the higher will be the risk. This contrasts with the results of a previous study conducted in long-known focus areas for *D. immitis* high-challenge transmission (frequency > 20%). In those areas, the length of time the dogs lived in the focus did not increase the infection frequency, perhaps because the focus was established, and transmission was quick (7). Therefore, it may be inferred that Baixada Fluminense is an area where *D. immitis* transmission is a recent event as a possible result of global environmental changes that demand extended periods of transmission (14).

In addition to the human population density, the presence of microfilaremic dogs (21) conditions the establishment of an enzootic cycle and the emergence of cases of human pulmonary dirofilariasis in areas of socio-environmental vulnerability, making it a worrying factor according to the One Health concept (22, 23). Therefore, implementing public policies for the management of environmental sanitation, control of vector mosquitoes by the endemic sector, and educational planning for health professionals by the local authorities is of paramount importance (24, 25). The study area once considered free of heartworm transmission, currently presents data suggesting the existence of this parasite (13). Therefore, once transmission in the area has been established, veterinarians must be prepared to guide pet owners to adhere to prevention and treatment measures.

With the occurrence of infected dogs in Baixada Fluminense documented herein, factors related to anthropogenic and climate, in addition to the presence of infected dogs (26, 27), may facilitate the establishment of competent mosquito populations and enhance the transmission of *D. immitis* in the region. Considering that Baixada Fluminense is a section of the state’s lowlands tangential to the oceanic coast and is a permanent conservation area, wild animals may also be affected (28). Most of the *D. immitis* infections documented in Brazil are in coastal areas (7, 29, 30), although infections are not restricted to these environments (31). The geographical dispersion of the parasite *D. immitis* in Brazilian previously indene regions are scarce (12, 13, 32, 33), however in Europe this spreading receives attention and is seen as a possible consequence of global climate changes (14, 34).

Thus, a broad epidemiological investigation must be conducted to monitor the prevalence of *D. immitis* in local dog populations by performing specific routine laboratory tests for detection and this filarioid identification. The rapid tests for antigen research are readily available and have greater specificity and sensitivity for the detection of *D. immitis* and to be recommended for clinical and epidemiological research (7, 30, 31).

The general recommendation is to request the modified Knott test (15) associated with antigen test (3) to detect and confirm parasitism in case of “occult infection” once 30% of the canine population will never be microfilaremic and because the predictive value of the antigen tests may provide false-positive results in a low-frequency area (30, 35).

## 5 Conclusion

The recently detected *D. immitis* infection in dogs in the lowland Baixada Fluminense region makes the area a candidate for canine heartworm transmission. This reinforces the need for an integrative approach among health professionals with a broad one-health perspective to implement public policies that promote health.

## Data availability statement

The raw data supporting the conclusions of this article will be made available by the authors, without undue reservation.

## Ethics statement

The animal studies were approved by Animal Use Ethics Committee of the Oswaldo Cruz Institute/ Oswaldo Cruz Foundation. The studies were conducted in accordance with the local legislation and institutional requirements. Written informed consent was obtained from the owners for the participation of their animals in this study.

## Author contributions

VA: Conceptualization, Data curation, Project administration, Investigation, Methodology, Writing – original draft. PS: Writing – original draft, Methodology. ÉP: Formal analysis, Methodology, Writing – original draft. PA: Methodology, Writing – original draft. NL: Methodology, Formal analysis, Writing – review & editing. GG: Methodology, Writing – review & editing, Conceptualization, Project administration, Supervision. AM: Conceptualization, Data curation, Project administration, Supervision, Writing – review & editing.

## Footnotes

^1^https://www.laborlife.com.br/portal/

^2^http://www.ceperj.rj.gov.br/
